# The Lesser Known Challenge of Climate Change: Thermal Variance and Sex-Reversal in Vertebrates with Temperature-Dependent Sex Determination

**DOI:** 10.1371/journal.pone.0018117

**Published:** 2011-03-23

**Authors:** Jennifer L. Neuwald, Nicole Valenzuela

**Affiliations:** Department of Ecology, Evolution and Organismal Biology, Iowa State University, Iowa, United States of America; Stanford University, United States of America

## Abstract

Climate change is expected to disrupt biological systems. Particularly susceptible are species with temperature-dependent sex determination (TSD), as in many reptiles. While the potentially devastating effect of rising mean temperatures on sex ratios in TSD species is appreciated, the consequences of increased thermal variance predicted to accompany climate change remain obscure. Surprisingly, no study has tested if the effect of thermal variance around high-temperatures (which are particularly relevant given climate change predictions) has the same or opposite effects as around lower temperatures. Here we show that sex ratios of the painted turtle (*Chrysemys picta*) were reversed as fluctuations increased around low *and* high unisexual mean-temperatures. Unexpectedly, the developmental and sexual responses around female-producing temperatures were decoupled in a more complex manner than around male-producing values. Our novel observations are not fully explained by existing ecological models of development and sex determination, and provide strong evidence that thermal fluctuations are critical for shaping the biological outcomes of climate change.

## Introduction

Climate helps determine many fundamental traits of organisms, from geographic distributions to life history patterns (e.g. [1]. Modifications of global and local biological patterns can thus be expected in response to climate change. Documented climatic-induced alterations of biological systems (e.g. [2], [3]) stress the urgency of understanding the effect of current and future climatic variation.

Specifically, changes in environmental temperature can profoundly alter the sex ratio of temperature-dependent sex determination (TSD) species, many of which are endangered. While most concern has focused on rising mean temperatures (e.g. [4], [5]), research on TSD reptiles indicates that natural sex ratios produced under daily temperature fluctuations may differ from those produced at constant incubation (e.g. [6], [7], [8], and references therein). Yet, the proximate TSD thermal mechanism remains unresolved. Notably, larger thermal fluctuations are predicted to accompany rising mean temperatures under climate change among years and decades [9], as well as seasonally [10], the scale at which sexual development of many TSD vertebrates occurs. Thus, to fully unravel the consequences of the complex thermal inputs experienced by TSD species, the full spectrum of ecologically-relevant temperatures and variation requires investigation. Previous work found that increasing the variance around low (male-producing) or intermediate (mixed-sex) temperatures feminized TSD turtle sex ratios [6], [11], [12], [13]. However, whether a similar variance experienced around high (female-producing) temperature induces females, males, or is lethal remains untested experimentally. Thus, it is unclear if enhancing the thermal variance around both unisexual means has the same or opposite effects on sex ratios. Here we address this question, which is critical for understanding the impact of climate change as the frequency of higher temperatures and the variance around those values increases, using the emerging model TSD turtle, *Chrysemys picta* [14].

### Ecological models of sex determination

A persistent challenge to understanding sex ratio evolution in TSD species is the difficulty of predicting sex ratios from natural nests where temperature fluctuates daily and often unpredictably [7], [15]. Many models have been proposed to address this issue, the simplest of which is the use of the mean incubation temperature as the sole predictor of nest sex ratio (e.g. [7], [16], [17]), or the combined use of the mean and variance of nest temperature in a bivariate plot under a sum-of-squares criterion to best fit a line separating male- from female-biased sex ratios [18], [19]. Other models have taken into account the cumulative effect of temperature on sex ratio by using as a predictor the number of hours at or above the temperature that produces 1∶1 sex ratios during the thermosensitive period [16], [18], [19], [20], [21], [22]. These models proved to be poor predictors of sex ratio or only suitable to few species. More recent ecological models have been devised to account for cumulative and differential effects of lower and higher temperatures on developmental rate and sex determination [6], [7], [23], [24], [25]. The most corroborated Constant Temperature Equivalent (CTE) model measures the proportion of development occurring above the threshold temperature, and predicts that fluctuations with constant variance about a stationary mean produce equal sex ratios as a constant temperature (i.e. CTE value) [6]. Second, a Cumulative Temperature Units (CTU) model accounts for temperature fluctuations by measuring the integrated time that the embryos spend above a biological threshold in a manner akin to a degree-day model [7]. However, the applicability of both models is restricted to temperatures within the range of values that have a linear effect on development [23], whereas temperatures experienced in natural nests and used in this study include values above and below this optimal temperature range (OTR) [8], [12], [26], [27], [28]. Finally, the latest is a variable degree model [25] developed for our study species, *Chrysemys picta*, which predicts the sex ratio of the nest as 100% male if the highest cumulative development over the thermosensitive period occurred within 22–28°C, 100% female if it occurred exclusively below 22°C or above 28°C, or 50% each sex if it occurred within 21–23°C or within 27.5–28.5°C. Thus, this model assumes that *C. picta* exhibits a low female-male threshold, an early conclusion that has been refuted by empirical data [29]. Additionally, this model's predictions are limited to trimodal sex ratios (0, 50, and 100% females) rather than accounting for the continuous sex ratios observed in nature.

To bypass the caveats described above and to account for the effect of temperatures outside the OTR, here we fit a non-linear model of development by temperature [23], [30] to incubation data from our study combined with data for *Chrysemys picta* from the literature [12], [13], [31], [32], [33], [34]. Developmental rate expressed as percentage per day (*r_a_*) was thus calculated as 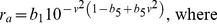
(1)




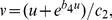
(2)




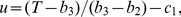
(3)

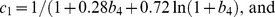
(4)




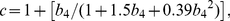
(5)where T represents the incubation temperature [23], [30]. The parameters describe the maximal developmental rate (*b_1_*) and the temperature (*b_3_*) at which it occurs, and *b_2_* corresponds to the temperature at which developmental rate is *b_1_*/10 [23], [30]. We then used this resulting developmental function (*r_a_*) to calculate the constant temperature predicted to induce a developmental rate equal to that observed in our fluctuating experiments (“*non-linear*” CTE values or nl-CTE), akin to the method used by the CTE model [6]. Implementation details are described in the methods section.

Our experimental approach reveals a more complex effect on developmental rate and sex ratio for the higher temperature regimes than previously anticipated, opening challenging questions about the potential response of TSD systems under predicted climate change.

## Methods

### Ethics Statement

All animal procedures were approved by Iowa State University IACUC under protocol # 5-05-5902-J.

**Figure 1:**
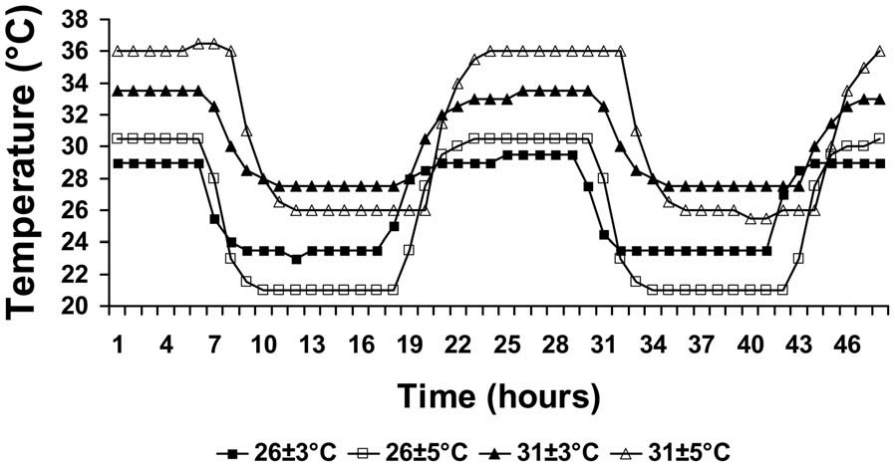
Two-day trace of the thermal regimes used in this study to incubate *Chrysemys picta* eggs.

Freshly-laid eggs of *Chrysemys picta* were incubated in moistened-sand [35] distributed among incubators set to fluctuate ±3°C and ±5°C around male−(26°C) and female−(31°C) producing means (Fig. 1, Table 1) [36]. Incubation temperatures included values that are experienced by natural nests [8], [26], [27], [28], the higher of which are predicted to become more frequent as mean temperature and variance increase with climate change [9], [10]. Daily temperature ranges used in our study are also within those experienced by nests in the field [37], [38]. Treatments minimized ramping time between temperatures as part of a parallel gene expression study to better disentangle the effect of mean versus thermal variance, and of high versus low temperature on sex ratios (Fig. 1). Twenty out of 93–123 eggs incubated per treatment were targeted for hatching for this study. Individuals were sexed by gonadal inspection 2.5–3.5 months post-hatching [36].

**Table 1:** Incubation experimental design used in this study of *Chrysemys picta*, and incubation parameters calculated from a non-linear model of development by temperature [23], [30], linear models [CTE (Georges et al. 1994) and CTU (Valenzuela 2001) models], and a variable degree model [25] of sex determination as described in the text.

	Incubation Treatment			
	26±3°C	26±5°C	31±3°C	31±5°C
**Minimum temperature (** **°** **C)**	23	21	28	26
**Maximum temperature (** **°** **C)**	29	31	34	36
**Mean temperature (** **°** **C)**	26	26	31	31
**nl-CTE** (i.e. constant temperature value predicted to produce an equivalent developmental rate as the fluctuating profiles, determined from the non-linear model)	26.2	25.6	28.9	27.2
**CTE** (i.e. constant temperature value predicted to produce an equivalent development per treatment as the fluctuating profiles)	26.7	28.0	31.6	32.5
**Daily CTU** from thermal traces (integral of hourly temperature records above developmental threshold of 14°C)	595	559	784	814
**VDM temperature** (i.e. temperature at which the highest cumulative development over the thermosensitive period occurred)	29	31	≥28	26

CTE and CTU units were calculated using 14°C as the developmental zero [33]. VDM temperature was calculated as 29°C and 31°C, respectively, for the 26±3 and 26±5°C treatments, and as 26°C for the 31±5°C treatment, since the minimum temperature in the former two treatments retards development compared to the maximum values while the opposite is true for the 31±5°C treatment. VDM temperature was calculated as ≥28°C for the 31±3°C treatment since embryos were exposed to temperatures between 28 and 34°C.

Sex ratios were compared to those from constant temperatures (i.e. ±0°C) to elucidate the effect of increasing amplitude of thermal fluctuations with respect to the effect of mean temperature [6], [11], [12], [13], [23], [33]. Deviations from expected sex ratios across treatments (100% males and 100% females from a 26°C and 31°C mean, respectively [36]) were evaluated using chi-square tests. The expected value for female-producing temperatures (frequency  =  0), was replaced by frequency  =  1 to avoid division by zero.

As mentioned before, natural nest temperatures and those used in our incubation experiments include values above and below the optimal temperature range (OTR) [8], [12], [26], [27], [28]. The non-linear model of development by temperature [23], [30] described by equations 1–5 above was fitted to *Chrysemys picta* incubation data for from our study and others [12], [13], [31], [32], [33], [34] using DEVARA software [30]. Temperature input data for the model included thermal profiles with constant or fluctuating temperatures and their corresponding observed incubation period (days) (this study and [12], [13], [31], [32], [33], [34]). Three of the constant parameters of the model (*b_1_–b_3_*) were obtained from the literature, while the remaining two parameters (*b_4_* and *b_5_*) were determined by iterative fitting as implemented in DEVARA using existing developmental data from our study and the literature. The parameters' values were: *b_1_* = 2.2 (maximal developmental rate), which occurs at *b_3_* = 32°C [32], and *b_2_* = 15.5 (temperature at which developmental rate is *b_1_*/10), which was calculated from the linear relationship of constant temperature and developmental rate for reported values within the OTR [12], [13], [31], [32], [33], [34]. The constant temperature predicted to induce a developmental rate equal to that observed in our fluctuating experiments (“*non-linear*” CTE values or nl-CTE) (Table 1) was calculated from *r_a_*. Additionally, we compared the results from this analysis with the results from the simpler CTE and CTU models [6], [7], as well as the VDM [25] to examine the discrepancies among model predictions.

Embryonic mortality was measured as the percent of eggs that died during incubation from the total number of eggs in each treatment. G-tests of independence were used to test for a temperature treatment effect in embryonic mortality. Incubation time was measured as days to hatching. The effect of temperature treatment on incubation length was tested using a Tukey-Kramer HDS test.

## Results

**Figure 2:**
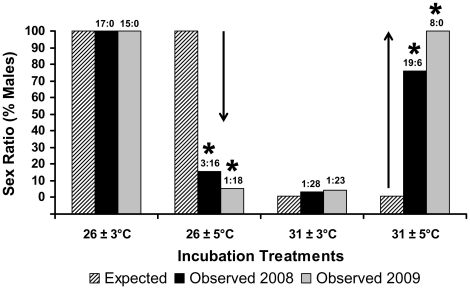
Reversing effect of increasing thermal variance on sex ratios of *Chrysemys picta* turtles. Observed males and observed females (m:f) per treatment and year are indicated above columns. Asterisks indicate statistically significant deviations from sex ratios expected by the mean temperature. Arrows indicate direction of change from expectation.

The experiment was replicated in 2008 and 2009 and produced consistent results (Fig. 2; χ^2^ = 5.8, df = 3, P>0.12; Table 2). Augmenting the variance around each unisexual mean reversed the sex ratios from those yielded by constant temperatures (Fig. 2). Sex ratios deviated significantly from expectation (χ^2^ = 337.5, 66.1, and 724.4 for 2008, 2009, and across years, respectively; df = 1; P<0.0001). Results were robust to removing cells with expected values of zero (χ^2^ = 13.5, 17.1, and 47.4 for 2008, 2009, and across years, respectively; df = 1; P<0.0002). Additionally, all sex ratios differed from parity (P<0.0001). Thus, while greater variance around low and intermediate mean temperature had a feminizing effect in this and other studies [6], [11], [12], [13], larger fluctuations around the high female-producing mean induced male differentiation. Mortality was significantly-higher under 31±5°C than under other treatments on both years (Table 3). Final sample sizes varied among treatments (Fig. 1) due to mortality or because eggs were diverted to, or added from, the concurrent gene expression study.

**Table 2:** Observed sex ratios of *Chrysemys picta*, and predictions from a non-linear model of development by temperature [23], [30], linear models [CTE (Georges et al. 1994) and CTU (Valenzuela 2001) models], and a variable degree model [25] of sex determination as described in the text.

	Incubation Treatment			
	26±3°C	26±5°C	31±3°C	31±5°C
**Mean temperature** **(**°**C)**	26	26	31	31
**Observed sex ratio (% male) 2008**	100§	15.8* † §	3.4†	76* † §
**Observed sex ratio (% male) 2009**	100§	5.3* † §	4.2†	100* † §
**Overall observed % male**	100§	8.8* † §	5.9†	81.8* † §
**Expected sex ratio given the mean temperature**	100	100	0	0
**Sex ratio (% male) predicted by the CTE values**	100	≈50	0	0
**Sex ratio (% male) predicted by the nl-CTE values**	100	100	≤50	100
**Sex ratio (%male) predicted by the VDM values**	0	0	0	100
**CTU-predicted (** ***and observed*** **) sex ratio order from 100% male ** [**1**] ** to 100% female ** [**4**]	2 (*1*)	1 (*3*)	3 (*4*)	4 (*2*)

* =  observed sex ratios unexplained by the mean temperature,

§ =  observed sex ratios unexplained by the linear and the VDM models,

† =  observed sex ratios unexplained by the non-linear model.

**Table 3:** Observed developmental rate and mortality of *Chrysemys picta* in this study.

	Incubation Treatment			
	26±3°C	26±5°C	31±3°C	31±5°C
**Mean ** ***(StDev)*** ** incubation time 2008** ‡ (days to hatching). Incubation time differed significantly among treatments with different letter superscript (Tukey-Kramer test, experimentwise α = 0.05).	70^A^ *(2.2)*	70^A^ *(1.9)*	56^B^ *(1.5)*	63^C^ *(3.5)*
**Mean ** ***(StDev)*** ** incubation time 2009** (days to hatching). Incubation time differed significantly among all treatments (Tukey-Kramer test, experimentwise α = 0.05).	64^A^ *(2.9)*	68^B^ *(4.1)*	51^C^ *(2.7)*	58^D^ *(1.5)*
**Embryonic mortality (%) 2008** ‡. ^§^Mortality statistically higher than other treatments (χ^2^ = 10.1, df = 3, P = 0.02 overall, versus χ^2^ = 2.8, df = 2, P = 0.24 when excluding 31±5°C).	7.53	10.64	14.95	22.22^§^
**Embryonic mortality (%) 2009**. ^§^Mortality statistically higher than other treatments (χ^2^ = 16.8, df = 3, P = 0.0008 overall, versus χ^2^ = 1.1, df = 2, P = 0.6 when excluding 31±5°C).	7.14	3.88	4.92	18.52^§^

Tukey-Kramer HSD significant differences in incubation time are indicated by the lettered superscripts.

‡ =  2008 values of mortality and incubation time are higher than in 2009 due to logistical problems with data-recording and thus are presented only to illustrate the relative effect of thermal treatments which is consistent across years, but not their absolute magnitude.

**Figure 3:**
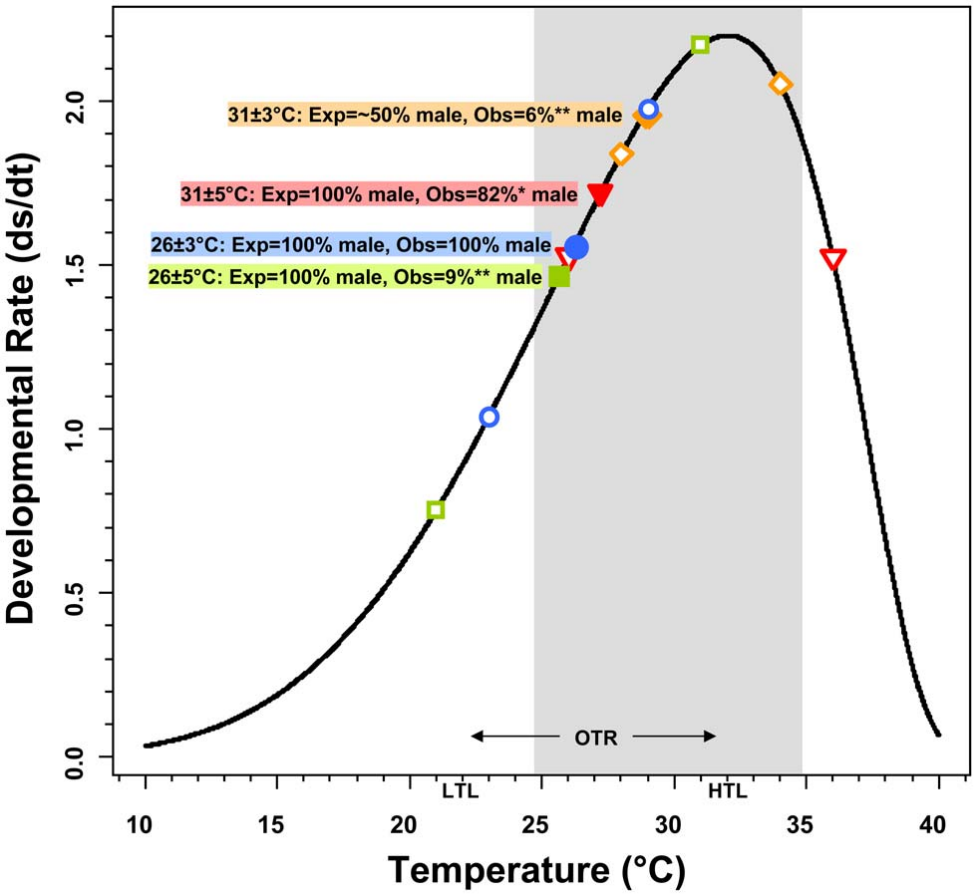
Developmental rate of *Chrysemys picta* embryos as a function of temperature. Solid symbols denote the constant temperature predicted by the non-linear model to exhibit a developmental rate equal to that observed at each fluctuating experiment conducted in this study (nl-CTE, see text for full description). Open symbols denote the developmental rate predicted for the minimum and maximum temperatures used in each fluctuating experiment. Symbols of the same shape and color correspond to a single fluctuating experiment as described in the color legends. Exp  =  sex ratio expected by the nl-CTE values. Obs  =  observed sex ratio. Asterisks denote deviations from expectation. OTR  =  Optimal Thermal Range (gray area), LTL  =  Low Thermal Limit, HTL  =  High Thermal Limit [12].

Not surprisingly, existing linear models of fluctuating temperature effects on development and sex determination [6], [7], [25] do not account for our results (Table 2). Indeed, the non-linear model fitted to the combined data from our study and from constant and fluctuating temperature experiments reported in the literature confirmed that 21°C (the minimum temperature in the 26±5°C treatment), 34°C and 36°C (the maximum temperatures in the 31±3°C and 31±5°C treatments, respectively) fall outside the OTR for *C. picta* of ∼22–32°C [12], and thus, have a retarding effect on developmental rate (Fig. 3). Accordingly, although incubation time was shorter overall in the 31°C-mean experiments than in the 26°C-mean experiments, development was slower under the ±5°C treatments as compared to the ±3°C treatments for each mean temperature (Table 3).

Notably, our results indicate that while increasing the variance around the male producing mean (26°C) had little effect in developmental rate, sex ratio was decoupled from the thermal effect on development under the largest fluctuations, whereas around the female producing mean (31°C) both the developmental rate and the sex ratios were affected by the thermal variance experienced. This unexpected observation reveals that the effect of increasing thermal fluctuations on sex determination depends upon the region of the temperature range where they fall. For instance, both the 26±3°C and 26±5°C treatments exhibited a developmental rate similar to that predicted by the non-linear model for a 26°C constant-temperature-equivalent (Table 2; Fig. 3), which should produce 100% males as observed for the 26±3°C experiment. The 26±°C treatment however, yielded 9% males instead of the predicted 100% males (Table 2). On the other hand, the 31±3°C treatment was expected to have produced ≥50% females as would a constant 28.9°C equivalent rather than the 6% males obtained. Further, the non-linear model predicted 31±5°C to have produced 100% males as would a constant 27.2°C rather than the 82% males obtained (Table 2).

## Discussion

We found that greater thermal variance around both low and high unisexual mean temperatures reversed the sex ratios of the painted turtle from those expected by the mean alone. Importantly, our results reveal that all levels of thermal fluctuation decouple developmental rate and sex ratios if experienced around the female-producing mean while this decoupling was only observed under the largest fluctuation around the male-producing mean temperature. Such decoupling means that the effect of temperature on sex ratio is not perfectly predicted by current models based solely on the effect that temperature has on development, revealing that the potency that temperature has to influence developmental rate differs somewhat from its potency to induce sex determination as described below.

### Thermal variance, development and sex determination

Despite our findings not matching predictions from the linear and non-linear ecological models, these patterns may nonetheless be explained by heat accumulation theory [6], [39]. Indeed, the high-variance treatments included temperatures that fall outside the OTR for *C. picta* [12] but which sustained development, which is consistent with theoretical expectations from and empirical observations in other TSD taxa [39]. Namely, embryos under 26±5°C (a treatment which produced sex ratios counter to expected) cycled between a 21°C minimum, a value below the lower thermal limit for this species [12] that slows down development (Fig. 3), and a 31°C maximum, a high female-producing value in the optimal range [12] (Fig. 3), such that greater embryonic development likely occurred under the female-producing temperatures despite the male-producing mean (Fig. 1). Similar to the 26±5°C treatment, embryos under 31±5°C cycled between 26°C (male-producing) and 36°C, a value above the upper thermal limit which retards growth (Fig. 3), such that development occurred mostly under the male-producing temperatures despite the female-producing mean. Consistently, incubation time was shorter for ±3°C than for ±5°C treatments (Table 3). Additionally, while mortality rates (3.5–22.2%) were commensurate with wild nests [40]), 31±5°C induced significantly higher mortality than other treatments (Table 3). Interestingly, embryos under 31±3°C cycled between 28°C (a value near the pivotal temperature that produces 1∶1 sex ratios) and 34°C, a value above the OTR which appeared to retard growth as expected (Fig. 3) while at the same time, exhibiting a greater potency than the lower temperature in inducing females (the sex expected by mean temperature). Thus, a highly female-biased sex ratio not significantly different from that predicted by the mean temperature alone was produced (Fig. 2).

The alternative that 21°C may induce female-differentiation in *C. picta* [21], [41] as low temperatures do in TSDII species (in which females are produced at both low and high temperatures, while males are produced at intermediate temperatures), was ruled out by extensive experimental data [29]). Furthermore, the variable degree model which assumes a TSDII model for *C. picta* [25] predicted that all treatments should produce 100% females except 31±5°C which should produce 100% males (Tables 1 and 2), an expectation far from our observed sex ratios. It is worth noting that while moisture was kept constant in our study and it may vary in the field, previous research has demonstrated that moisture levels do not affect sex determination in *C. picta* [42].

Our results emphasize that a general model accounting for both mean and variance across the full range of viable temperatures remains overdue to explain sex determination and accurately forecast sex ratios under climate change. Importantly, we show for the first time that the amplitude of thermal fluctuations mediate the sex ratio response to mean temperature around female-producing values in a more complex way than it does around male-producing values ([6], [11], [12], [13], [33], and this study). Thermal values and fluctuations used in this experiment are within the range experienced by nests of this species in the field [8], [26], [27], [28], [37], [38]. Thus, while the 31°C-mean treatments had a higher mean than the averages recorded in natural populations, our design permitted testing the tolerance of this species to long exposure to high temperatures that are already experienced in the wild and which may be encountered more frequently in the future if mean and variance increase due to climate change as currently predicted [10]. Therefore, our experimental design helped place the female-producing temperature used broadly in laboratory studies (e.g. [36], [43], [44]) in the same context as the better-studied male-producing and intermediate temperatures [6], [11], [12], [13], [33]). Our novel observations reveal that the effect of increasing thermal fluctuations on sex determination depends upon the region of the temperature range where they fall, consistent with reports for other phenotypes in TSD and GSD taxa [12], [45], [46]. Furthermore, our results open the question about whether the effects of temperature mean and variance on multiple traits in other biological systems may be decoupled as observed here in ways that have not been previously anticipated.

### Climate change and TSD evolution

Our results strengthen the concern about the fate of TSD systems facing chronic environmental disturbances (e.g. [6], [7], [8], and references therein). Variance in continental temperature is expected to increase during the summer [10] when air temperatures influence sex ratios of wild *C. picta* [4]. Although potential negative effects of climate change might be lessened by compensatory plastic or rapid evolutionary responses [6], these may be constrained in endangered TSD taxa under low population sizes or disturbed habitats. At first glance, our observations would misleadingly suggest that if thermal variance increases during the reproductive season as current climate change models predict [10], the sex-reversing effect of greater fluctuations would be beneficial by helping buffer against the effect of changes in the temperature mean alone. Furthermore, a recent study in *C. picta* showed that increasing the thermal variance around an intermediate temperature had no effect in hatchling morphological, behavioral or immunological phenotypes, nor in embryonic mortality [13] thus, ruling out several potential negative effect of thermal variance at lower temperatures. However, whether phenotypic responses differ under larger thermal fluctuations around higher mean temperatures remains untested, and our data indicate that at least mortality is higher under those conditions. Notably therefore, our findings suggest that higher embryonic mortality may offset any benefit accrued by the masculinizing effect of higher variance around female-producing mean temperatures, and may consequently interact with other factors that mediate the effect of a thermally-changing world. For instance, nesting behavior has been proposed as another potential compensatory response to climate change given that canopy openness affects the daily temperature range (a proxy of thermal variance) and consequently the sex ratios in TSD species [47]. The sex-reversing effect of thermal variance observed in our study would superficially suggest that shallow nesting TSD species, such as *C. picta*, may be more able to respond to climate change than deeper nesting taxa, such as sea turtles [6]. However, larger variation resulting from a compensatory nesting response in *C. picta* might expose the shallower eggs to lethally- or suboptimaly-high temperatures, as occurred in our study and in other species (e.g. [48]).

**Figure 4:**
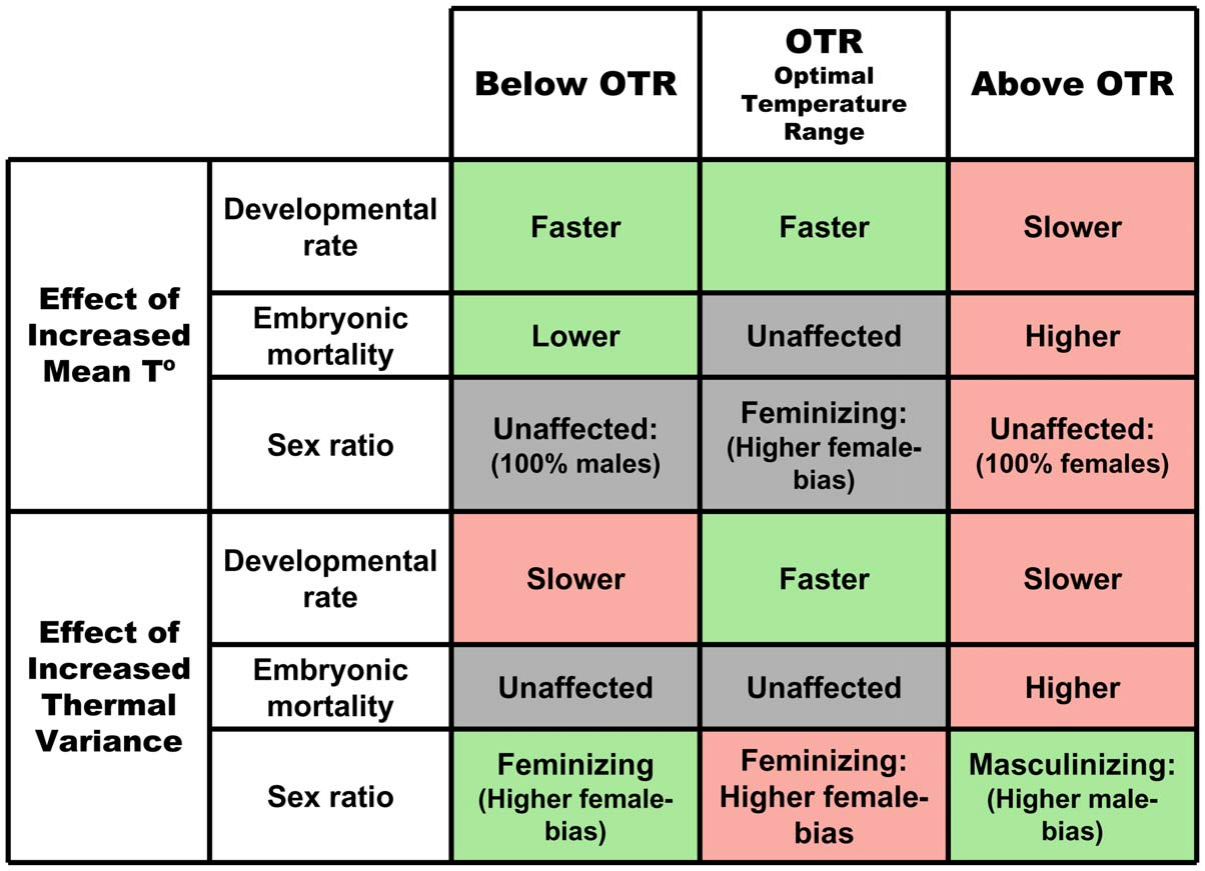
Observed effects of increased mean temperature and increased thermal variance on life history parameters of the TSDIa turtle, *Chrysemys picta*, and implications in the context of climate change predictions. Effects are divided into three thermal ranges: optimal temperatures (OTR), colder temperatures below the OTR, and warmer temperatures above the OTR. Inner cells correspond to neutral effects (gray), beneficial effects (green), and detrimental effects (pink) on developmental rate, embryonic survival, and sex ratio, as described in the text. Listed effects correspond to those of increased mean temperature alone under low or no variance scenarios, and to those of increased thermal variance when compared to mean temperature effects.

Fig. 4 summarizes our findings in the context of climate change for *Chrysemys picta*, a turtle exhibiting a TSDIa pattern of sex determination (where males are produced at low temperatures and females at high)[49], but the same logic can be extended in the opposite direction for TSDIb taxa (where females are produced at low temperatures and males at high) or TSDII taxa (where males are produced at intermediate temperatures and females above and below these values) since global warming will affect them in a similar way as for TSDIa taxa. Under limited thermal variance, increases in mean temperatures below the optimal temperature range (OTR) accelerate development and improve survival as values move closer to the OTR, although these values are exclusively male-producing (Fig. 4). Within the OTR, higher mean temperatures also accelerate development but mortality is unaffected since this range is optimal, and the proportion of females increases as a logistic function of the mean temperature from all-male- to all-female- producing (Fig. 4)[49]. One detrimental effect of climate change derives from causing TSDIa taxa to produce excessively female-biased sex ratio that endangered population persistence due to male-limitation (or female limitation due to excessively male-biased sex ratio in the case of TSDIb taxa). Under these low thermal variance conditions above the OTR, higher temperatures reach values that are detrimental for development and survival but remain all-female-producing (Fig. 4). The combination of extreme biased sex ratios as described earlier and increased mortality represent the dangers of global warming at these values. In contrast, as thermal variance increases below the OTR, embryos are exposed to lower suboptimal temperatures that slow down development but do not affect mortality (if they remain mainly above lethal values), such that most development occurs at the higher temperatures which thus tend to produce higher female-biases compare to the mean temperature (Fig. 4). This feminizing effect will be beneficial for a TSDIa population suffering from excessively male-biased sex ratios. Within the OTR, increasing the thermal variance exposes the embryos to higher optimal values that accelerate development and have a feminizing effect, and which exhibit more potency to stimulate development than lower values to slow down development and affect sex ratio. These temperatures within the OTR do not affect mortality (Fig. 4). Thus, within the OTR, the potential danger of increased variance due to climate change would be the production of excessively female-biased sex ratios in TSDIa taxa. Above the OTR, increasing the thermal variance exposes embryos to excessively high temperatures that inhibit development and cause higher mortality, such that development occurs mostly at the lower temperatures resulting in a net masculinizing effect (Fig. 4). This masculinizing effect would be beneficial to counter the female-biases induced by higher mean temperatures under climate change, but these benefits may be offset by the higher mortality suffered which may lead to severe population bottlenecks.

Further research is warranted to explore the effect of increased variance around actual natural thermal profiles on sex and fitness to test more realistic scenarios of sex determination and environmental perturbations. Additionally, studies to reveal the molecular mechanism responsible for our observations are urgently needed. Both research studies are ongoing. Whether greater fluctuations in the wild have the same effect as those observed here and around intermediate temperatures [13] requires investigating the interaction between thermal variance and other biological traits, including incubation length, nesting timing, generation time, and TSD pattern, among others. For instance, turtles and lizards with contrasting TSD patterns may respond differently to such changes [49]. Importantly, if TSD is an adaptive trait (*sensu* [50]), an increased thermal variance may decouple the environmental variables that confer sex-specific fitness at different temperatures throughout the reproductive season or geographic locations, potentially breaking down the adaptiveness of this sex-determining mechanism, and perhaps even inducing a transition in sex determining mechanisms. Interestingly, such transitions between TSD and GSD systems over 200 my of turtle evolution are associated with dramatic genomic rearrangements and appear to coincide with climate change events [51]. Alternatively, the untested hypothesis that thermal variance itself induces differential fitness, or is correlated with such a variable [50], and therefore underlies the evolution or maintenance of TSD [15], should be considered. A recent initial study in *C. picta* suggested that sex and other phenotypic responses to thermal variance are decoupled at least for embryonic and hatchling life stages [13], but research on other systems demonstrates that responses to incubation conditions may be delayed to the reproductive life stages [52].

In summary, our study underlines the importance of investigating the role of thermal variance to understand TSD sex ratio evolution, its consequences, and its effect on other fitness-relevant phenotypes to understand the response of biodiversity to local and global disturbances at multiple time scales.
